# Determination of B and T Cell Epitopes in *Neospora caninum* Immune Mapped Protein-1 (IMP-1): Implications in Vaccine Design against Neosporosis

**DOI:** 10.1155/2022/2508050

**Published:** 2022-04-07

**Authors:** Naser Nazari, Bahareh Kordi, Bahman Maleki, Morteza Shams, Esfandiar Azizi, Hamidreza Majidiani, Razi Naserifar

**Affiliations:** ^1^Department of Parasitology and Mycology, School of Medicine, Kermanshah University of Medical Sciences, Kermanshah, Iran; ^2^Department of Basic Medical Sciences, Neyshabur University of Medical Sciences, Neyshabur, Iran; ^3^Department of Parasitology, Faculty of Medical Sciences, Tarbiat Modares University, Tehran, Iran; ^4^Zoonotic Diseases Research Center, Ilam University of Medical Sciences, Ilam, Iran; ^5^Department of Immunology, Faculty of Medicine, Ilam University of Medical Sciences, Ilam, Iran

## Abstract

Prevention of neosporosis is advantageous for cattle health and productivity. Previously, several vaccine candidates were nominated for vaccination against *Neospora caninum*. This study was premised on *in silico* evaluation of *N. caninum* IMP-1 in order to determine its physicochemical features and immunogenic epitopes. We employed a wide array of network-based tools for the prediction of antigenicity, allergenicity, solubility, posttranslational modification (PTM) sites, physicochemical properties, transmembrane domains and signal peptide, secondary and tertiary structures, and intrinsically disordered regions. Also, prediction and screening of potential continuous B cell peptides and those epitopes having stringent affinity to couple with mouse major histocompatibility complex (MHC) and cytotoxic T lymphocyte (CTL) receptors were accomplished. The protein had 393 residues with a molecular weight of 42.71 kDa, representing aliphatic index of 85.83 (thermotolerant) and GRAVY score of -0.447 (hydrophilic). There were 47 PTM sites without a signal peptide in the sequence. Secondary structure comprised mostly of extended strand and helices, followed by coils. The Ramachandran plot of the refined model showed 90.1%, 9.9%, 0.0%, and 0.0% residues in the favored, additional allowed, generously allowed, and disallowed regions, correspondingly. Additionally, various potential B cell (linear and conformational), CTL, and MHC binding epitopes were predicted for *N. caninum* IMP-1. The findings of the present study could be further directed for next-generation vaccine design against neosporosis.

## 1. Introduction

Neosporosis is a parasitic disease caused by an intracellular apicomplexan, *Neospora caninum* (*N. caninum*) [1], with serious sequelae such as reproductive failure in livestock species, particularly in cows [2, 3]. This protozoan also infects rodents, wild ungulates, birds, and marine mammals [4]. The parasite employs two hosts to complete its life cycle, so that dog (*Canis familiaris*) [5], dingo (*Canis dingo*) [6], coyote (*Canis latrans*) [7], and gray wolf (*Canis lupus*) [8] are definitive hosts, while cattle and buffalo are the most important intermediate hosts [9]. The parasite possesses three distinct infective stages, comprising tachyzoite (acute infection), bradyzoite (chronic infection), and sporozoite (environmental contamination) [10]. Infected canids contaminate the environment through oocyst shedding, being infectious for both canids and herbivores [11]. It is estimated that *N. caninum* infections waste over US$1 billion annually in both beef and dairy cattle industries [12]. The parasite is maintained within cattle populations through transplacental transmission, resulting from oocyst ingestion (exogenously) and/or reactivated infection during gestation (endogenously) [13, 14]. In addition to the endemic and/or epidemic abortions in midgestation, there are other factors that economically impact the cattle industry including reduced weight gain in beef calves, decreased milk yield [10], replacing culled animals [15], and the additional costs of veterinary care [16].

Ordinarily, various strategies are proposed to cattle producers in order to reduce infections within herds, including (i) identify and cull infected animals in case of endemic abortions; (ii) prevention of contact between cattle and definitive hosts, hence reducing oocyst contamination, in case of epidemic abortions; (iii) chemotherapy of seropositive animals; and (iv) vaccination protocols [17]. Lack of effective and safe drugs, on the one hand, and long-time treatment causing the issue of drug residues in food animals, on the other hand, make treatment troublesome economically [15, 18]. Thereby, vaccination strategies sound more economic sense to impede the infection [19]. Despite over a decade of research on immunization against *N. caninum* using various protocols, no commercial vaccine has been developed so far [20]. An ideal vaccination against *N. caninum* may comply with several issues, encompassing a considerable decline in oocyst shedding by final hosts, reduction of tissue cysts in food animals to avoid transmission *via* carnivorism, and confining tachyzoite multiplication in pregnant cow to lower the rate of transplacental transmission [17]. Accordingly, such vaccine candidate should stimulate both mucosal and systemic cell-mediated and antibody-dependent components [21]. Thus far, several vaccination strategies using naturally less-virulent isolates and/or attenuated strains have been exploited in cattle and mouse models, showing to be efficacious in spite of safety concerns and production costs [10]. Subunit peptide-based or DNA vaccines are more deeply investigated due to their evident benefits in reduced production, processing, and storage costs along with higher shelf-life and stability [22]. Mostly, those molecules involved in adhesion/invasion processes such as surface antigens (SAGs), microneme (MIC), and rhoptry (ROP) proteins, dense granular (GRA) components, and targets in parasitophorous vacuole membrane (PVM) have been targeted in subunit vaccines [23].

Immunoinformatics is an emerging computer-aided practice for a rational, structure-based vaccine design in a time- and cost-effective manner, which also optimizes biochemical and immunogenic performances [24]. Previously, *N. caninum* immune mapped protein-1 (NcIMP-1) was shown as one of the promising vaccine candidate [25]. Nevertheless, lack of information on biochemical features and potential immunogenic epitopes in mouse models directed us to conduct the present study *in silico* study, being beneficial for future vaccine research on neosporosis.

## 2. Methods

### 2.1. NcIMP-1 Protein Sequence Retrieval

The amino acid sequence of the NcIMP-1 protein was retrieved through the UniProtKB database, available at https://www.uniprot.org/, under accession number of J9PWX7.

### 2.2. Prediction of Antigenicity, Allergenicity, Solubility, and Physicochemical Characteristics

Antigenicity is a principal characteristic of a vaccine candidate and was evaluated using two web servers: ANTIGENpro (http://scratch.proteomics.ics.uci.edu/) and VaxiJen v2.0 (http://www.ddgpharmfac.net/vaxijen/). The latter is a freely accessible server which predicts on the basis of physicochemical properties of a protein and turns sequences into uniform vectors via the autocross-covariance (ACC) approach. Also, ANTIGENpro is a pathogen-independent, alignment-free predictor of antigenicity using a two-stage architecture and five ML algorithms, trained by reactivity information obtained from protein microarray analyses for five pathogens. Three web servers predicted allergenicity, including AlgPred (http://crdd.osdd.net/raghava/algpred/), AllergenFP v1.0 (https://ddgpharmfac.net/AllergenFP/), and AllerTOP v2.0 (http://www.ddg-pharmfac.net/AllerTOP). An alignment-free approach with the Mathews correlation coefficient of 0.759 is employed by AllergenFP v1.0 server, while AllerTOP v2.0 exploits several machine learning methods, comprising *k*-nearest neighbors, cross-variance transformation, and E-descriptors. Moreover, mapping IgE epitopes, MEME (Multiple Em for Motif Elicitation)/MAST (Motif Alignment and Search Tool) allergen motifs were utilized by AlgPred web server to predict allergens. Protein-Sol web server, available at https://proteinsol.manchester.ac.uk/, predicted solubility of NcIMP-1 with a threshold score of 0.45 as the population average of the experimental dataset, so higher scores indicate to higher protein solubility. Finally, ExPASy ProtParam server (https://web.expasy.org/protparam/) was used to estimate some important physicochemical properties of NcIMP-1 such as molecular weight (MW), number of negatively and positively charged residues, aliphatic and instability indices, isoelectric point (pI), half-life, and grand average of hydropathicity (GRAVY).

### 2.3. Prediction of Posttranslational Modification (PTM) Sites

Several PTM sites of NcIMP-1 protein were predicted, including serine, threonine, and tyrosine phosphorylation sites by NetPhos 3.1 (http://www.cbs.dtu.dk/services/NetPhos/), palmitoylation, or acylation sites by CSS-Palm (http://csspalm.biocuckoo.org/) as well as N-linked and O-linked glycosylation sites by NetNGlyc 1.0 (http://www.cbs.dtu.dk/services/NetNGlyc/) and NetOGlyc 4.0 (http://www.cbs.dtu.dk/services/NetOGlyc/) web servers. “All Asn residues” option was used for NetNGlyc 1.0 prediction, while default parameters were applied to NetOGlyc 4.0 server.

### 2.4. Signal Peptide and Transmembrane Domain Prediction

For transmembrane domain prediction, TMHMM 2.0 server was used, being available at http://www.cbs.dtu.dk/services/TMHMM-2.0. In the following, signal peptide prediction was done using two web servers, including Signal-3L 3.0 (http://www.csbio.sjtu.edu.cn/bioinf/Signal-3L/) and SignalP (http://www.cbs.dtu.dk/services/SignalP/) web servers.

### 2.5. Secondary Structure and Disordered Regions Prediction

Prediction of the secondary structure was done by the PSI-blast-based secondary structure PREDiction (PSIPRED) server, which is available at http://bioinf.cs.ucl.ac.uk/psipred/. This server shows many important features in the submitted protein sequence, if available, such as strand, helix, coil, disordered regions, putative domain boundary, membrane interaction, transmembrane helix, extracellular, reentrant helix, cytoplasmic, and signal peptide in both sequence-based and graphical forms.

### 2.6. Prediction of the Three-Dimensional (3D) Model, Refinement, and Validations

The homology modelling of the NcIMP-1 protein was performed using Swiss-Model online tool using default parameters (https://swissmodel.expasy.org/). In order to establish likely side chains, repacking them, and total refinement of the final structure, the GalaxyRefine server (http://galaxy.seoklab.org/cgi-bin/submit.cgi?type=REFINE) was used which provides five refined models for each submitted pdb file, differing on several parameters such as global distance test-high accuracy (GDT-HA), root mean square deviation (RMSD), MolProbity, Clash score, Poor rotamers, and Rama favored. Subsequently, the quality improvement of the final structure was evaluated using ERRAT (quality factor) and PROCHECK (Ramachandran plot analysis) (https://saves.mbi.ucla.edu/).

### 2.7. Prediction of Continuous and Conformational B Cell Epitopes

A multistep approach was exploited for linear B cell epitope prediction in NcIMP-1. For this aim, a fixed-length prediction (14-mer) with 75% specificity was applied in BCPred server (http://ailab.ist.psu.edu/bcpred/predict.html), which uses subsequent kernel (SSK) and support vector machine (SVM) techniques. In the next step, cross-validation of the predicted epitopes was accomplished with the outputs of two other web servers, including ABCpred (http://crdd.osdd.net/raghava/abcpred/ABC_submission) and SVMTriP (http://sysbio.unl.edu/SVMTriP/prediction.php). Those epitopes being shared among outputs of the above servers were selected for further screening regarding antigenicity, allergenicity, and water solubility using VaxiJen v2.0, AllerTOP v2.0, and PepCalc web servers, respectively. Of note, linear B cell epitopes were, also, predicted by BcePred server based on different physicochemical parameters such as hydrophobicity, flexibility, accessibility, turns, exposed surface, polarity, and antigenic propensity (http://crdd.osdd.net/raghava/bcepred/bcepred_submission.html). Additionally, conformational B cell epitopes were predicted using ElliPro tool of the immune epitope database (IEDB) web server (http://tools.iedb.org/ellipro/).

### 2.8. Prediction and Screening of Mouse Major Histocompatibility (MHC) Binding Epitopes

All epitope predictions were done using MHC-I (http://tools.iedb.org/mhci/) and MHC-II (http://tools.immuneepitope.org/mhcii) binding epitope prediction tools of IEDB server. Regarding MHC-I binding epitopes, 8 mouse alleles (H2-Db, H2-Dd, H2-Kb, H2-Kd, H2-Kk, H2-Ld, H-2-Qa1, and H-2-Qa2) were used with subsequent screening in terms of antigenicity, allergenicity, and toxicity through VaxiJen v2.0, AllergenFP v1.0, and ToxinPred (https://webs.iiitd.edu.in/raghava/toxinpred/index.html) servers, respectively. With respect to MHC-II binding epitopes, 3 mouse alleles (H2-IAb, H2-IAd, and H2-IEd) were employed for epitope prediction, followed by screening regarding antigenicity, allergenicity, toxicity, and IFN-*γ* and IL-4 induction using VaxiJen v2.0, AllergenFP v1.0, ToxinPred, IFNepitope (https://webs.iiitd.edu.in/raghava/ifnepitope/application.php), and IL4-pred (https://webs.iiitd.edu.in/raghava/il4pred/design.php) web servers, correspondingly.

### 2.9. Prediction and Screening of Cytotoxic T Lymphocyte (CTL) Epitopes

Top 10 CTL epitopes of NcIMP-1 protein were predicted using CTLPred web server (https://bio.tools/ctlpred), followed by screening regarding antigenicity, allergenicity, and hydrophobicity using VaxiJen v2.0, AllergenFP v1.0, and peptide2 (https://www.peptide2.com/N_peptide_hydrophobicity_hydrophilicity.php) web servers, respectively.

## 3. Results

### 3.1. General Characteristics of the NcIMP-1 Protein

**Figure 1:**
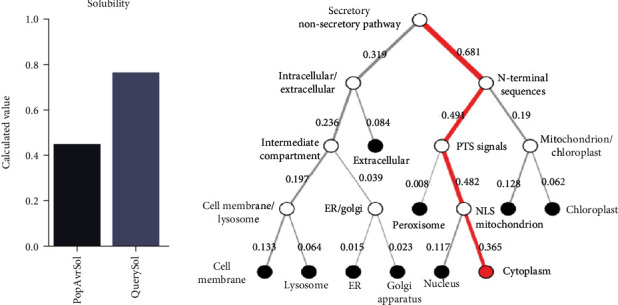
Computed solubility (a) and subcellular localization (b) of the NcIMP-1 protein.

A considerably high antigenic index was predicted for this protein, as substantiated by a VaxiJen score of 0.6613 and ANTIGENpro score of 0.838802. Based on the findings from three web servers, no allergenicity, IgE epitopes, and MEME/MAST motifs were found for NcIMP-1 protein. A considerably high solubility (over 0.45) was, also, predicted by Protein-Sol server with a solubility score of 0.764 (Figure 1). This protein possessed 393 amino acid residues, with a MW of 42717.22 kilodalton (kDa) and 63 and 53 negatively (Asp+Glu) and positively charged (Arg+Lys) residues. The extinction coefficients at 280 nm measured in water was 45045 (assuming all pairs form cystines) and 44920 M^−1^ cm^−1^ (assuming all Cys residues are reduced). The estimated half-life was 30 hours in mammalian reticulocytes (*in vitro*), >20 hours in yeast (*in vivo*), and >10 hours in *Escherichia coli* (*in vivo*). The protein was rendered as unstable, since instability index was computed to be 41.29. Moreover, aliphatic index and GRAVY score were 85.83 and -0.447, respectively. Of note, the calculated pI for this protein was relatively acidic (5.43).

### 3.2. Prediction of PTM Sites, Subcellular Localization, Transmembrane Domain, and Signal Peptide

In total, 33 phosphorylation sites were present in the NcIMP-1 protein using NetPhos server, encompassing 20 serine, 9 tyrosine, and 3 threonine sites. Also, a palmitoylation site at position 5 was found with a score of 39.402 using CSS-Palm server. In addition, NetOGlyc web server predicted 13 NO-glycosylation sites in the examined protein, while there was no N-glycosylation region in the sequence. No putative transmembrane domain was predicted for this protein, as demonstrated by the TMHMM server. Outputs of the Signal-3L server (reliability: 1.0) and SignalP (likelihood for others: 0.9986) web tools showed no traits of a signal peptide in NcIMP-1 protein. DeepLoc subcellular localization analysis revealed that NcIMP-1 is probably a soluble (likelihood: 0.4027), cell membrane protein (likelihood: 0.2081) with membrane localization (likelihood: 0.5973) (Figure 1).

### 3.3. Secondary Structure Prediction and Disordered Regions

**Figure 2:**
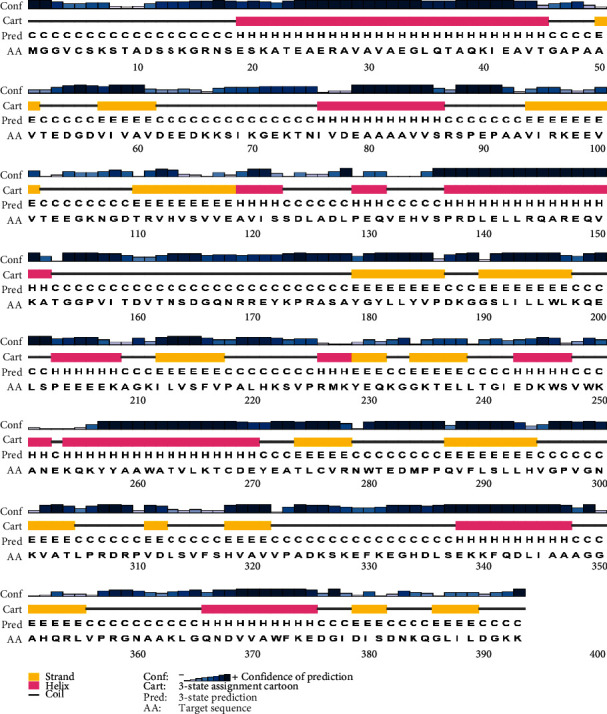
Secondary structure prediction by PSIPRED server showing the predominance of extended strand.

Based on the PSIPRED server analysis with high confidence in most parts, extended strand and helices were the most predominant secondary structures in the NcIMP-1 protein, followed by coils. Also, no intrinsically disordered regions were found in this protein. The graphical output of secondary structure prediction is provided in Figure 2.

### 3.4. 3D Structure Modelling, Refinement, and Validations

**Figure 3:**
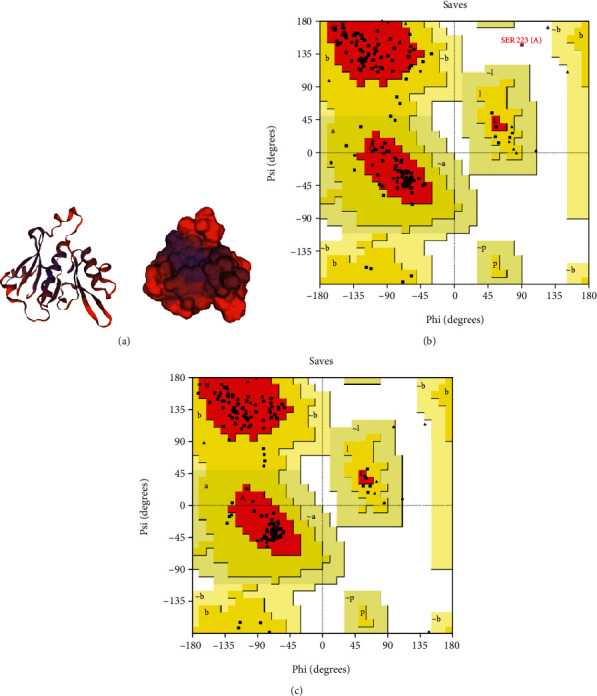
NcIMP-1 protein homology modelling and refinement validation using Ramachandran analysis. (a) The final tertiary model of NcIMP-1 provided by SWISS-MODEL web server, as shown in ribbon and surface. (b) Ramachandran plot analysis of the crude model using PROCHECK demonstrated 83.6%, 15.8%, 0.0%, and 0.7% of residues are assigned to most favored, additional allowed, generously allowed, and disallowed areas, respectively. (c) Upon refinement, they were improved to 90.1%, 9.9%, 0.0%, and 0.0%, correspondingly.

Three models were built by SWISS-MODEL server, among which a monomer model (template: 5lg9.1. A) with moderate coverage and sequence identity of 23.03% was selected for further analysis (Figure 3(a)). In the following, GalaxyRefine server provided five models, among which model number five with the following parameters was chosen as the best-fit refined model: GDT-HA: 0.9702, RMSD: 0.354, MolProbity: 1.951, Clash score: 19.9, Poor rotamers: 0.7, and Rama favored: 97.1. Finally, the quality of the refined model, as compared with the crude model, was evaluated using three web servers. The quality factor of the crude model was 83.234, which was improved to 85.976 after refinement, respectively. Ramachandran plot analysis of the crude model showed that 83.6%, 15.8%, 0.0%, and 0.7% of residues are assigned to most favored, additional allowed, generously allowed, and disallowed areas, respectively. Upon refinement, they were improved to 90.1%, 9.9%, 0.0%, and 0.0%, correspondingly (Figures 3(b) and 3(c)).

### 3.5. Linear and Conformational B Cell Epitopes

**Figure 4:**
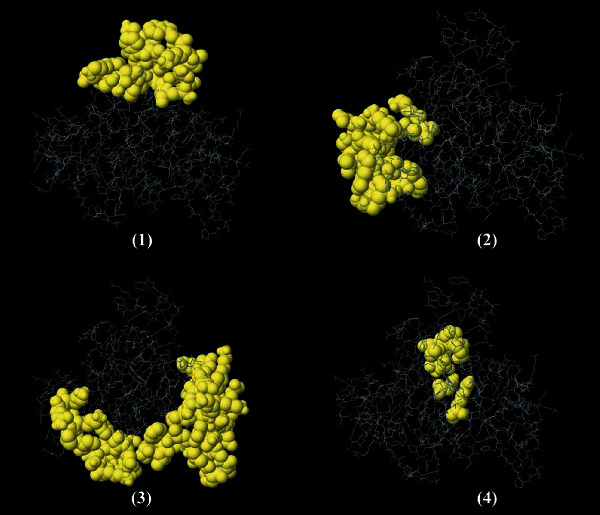
Predicted conformational B cell epitopes of NcIMP-1 using ElliPro tool of IEDB server. Length and score of each epitope were as follows: (1) 21 residues, score: 0.712; (2) 24 residues, score: 0.697; (3) 49 residues, score: 0.662; and (4) 8 residues, score: 0.557.

A cross-validating method was applied to find shared linear B cell epitopes. Accordingly, 8 epitopes were found and subsequent screening showed that only six epitopes are potentially antigenic, nonallergenic with good water solubility, including “VTEDGDVIVAVDE,” “TADSSKGRNSESK,” “MKYEQKGGKTE,” “KSIKGEKTNIV,” “STADSSKGRN,” and “EKAGKILVSFVPA” (Table 1). Moreover, several continuous B cell epitopes of NcIMP-1 protein were determined on the basis of various physicochemical parameters using BcePred web server (Table 2). Also, ElliPro tool of the IEDB analysis resource demonstrated that there are 4 conformational B cell epitopes in this protein with the following lengths and scores: (i) 21 residues, score: 0.712; (ii) 24 residues, score: 0.697; (iii) 49 residues, score: 0.662; and (iv) 8 residues, score: 0.557 (Figure 4).

### 3.6. Prediction of Mouse MHC Binding and CTL Epitopes

For each mouse MHC-I (H2-Db, H2-Dd, H2-Kb, H2-Kd, H2-Kk, H2-Ld, H-2-Qa1, and H-2-Qa2) and MHC-II allele (H2-IAb, H2-IAd, and H2-IEd), five and six epitopes having the lowest percentile rank (higher affinity) were chosen, respectively, which was then subjected to screening in terms of antigenicity, allergenicity, toxicity (MHC-I and MHC-II), and IFN-*γ*/IL-4 induction (MHC-II). Regarding mouse MHC-I binding epitopes, four epitopes had the highest antigenicity score, while they were nonallergen and nontoxic, including: “VDLSVFSHVAVV,” “EEEKAGKILVSF,” “LPRDRPVDLSVF,” and “DEYEATLCVRNW” (Table 3). Furthermore, nine mouse MHC-II binding epitopes were capable to induce IFN-*γ*, whereas they lacked the adequate antigenicity score (threshold 0.5). Of note, all epitopes were capable of inducing IL-4, except of “DLSVFSHVAVVPADK” peptide (Table 4). Also, top ten CTL epitopes were predicted using CTLPred server, among which only one epitope possessed highest antigenicity and hydrophobicity and without allergenicity, i.e., “QVKATGGPV” (Table 5).

## 4. Discussion

First insights into the immunobiology of the apicomplexan parasite, *N. caninum*, in cattle and dogs were revealed during 1999 to 2003 [20], leading to the initial vaccination approaches in the mouse model [26] as well as cattle as target species [27]. In parallel with the deciphering the parasite biology and identification of parasitic antigens, more researches on *N. caninum* vaccination were flourished during last decade, using novel antigens and different immunization platforms. Having no live component, subunit vaccines represent no risk of disease induction; hence, they are mostly focused for a safe vaccination, usually accompanied by an adjuvant as an immune promoter compound [24]. Innovative technology-oriented methods such as reverse vaccinology and immunomics have facilitated the appropriate screening and selection of potential antigenic targets among multiple proteins and assisted us to deeply explore and highlight the immunogenic epitopes within the amino acid sequence of a given protein [24]. Until now, several surface expressed and excretory/secretory proteins have been recognized as vaccine candidates [28–32], while *in silico* analysis of such proteins and identification of potential immunogenic epitopes was lacking. The present *in silico* study was performed to highlight several important biochemical properties of the NcIMP-1 protein and to identify novel immunogenic epitopes for future vaccination and/or diagnostic purposes in the context of multiepitope protein constructs.

This protein is probably highly conserved among apicomplexan parasites and initially recognized as a protective antigen in an important poultry parasite, *Eimeria maxima* [33]. In 2012, Cui et al. introduced NcIMP-1 protein as a novel membrane-bound molecule and showed that specific anti-NcIMP1 antibodies could substantially harness the tachyzoite invasion *in vitro* [30]. Further, in a vaccination study by [25], it was shown that immunized mouse with pcDNA-IMP-1 demonstrated mixed IgG1/IgG2a response, particularly IgG2a, upsurge of IFN-*γ*, IL-2, IL-4, and IL-10 and significant reduction in cerebral parasite burden [25]. Based on such findings, it can be speculated that this protein could be a potential vaccine candidate. “From a biochemical standpoint, a protein is represented in four structural levels, comprising (i) amino acid sequences as primary structure, (ii) a native spatial form due to main chain atoms (*α*-helix and *β*-fold) as secondary structure, (iii) potential spatial model as a 3D model or tertiary structure, and (iv) number and position of multifold subunits in a multisubunit collection of a protein as quaternary structure” [34–36]. In the first step of this study, we characterized general biochemical features of the protein. It was found that NcIMP-1 is a highly antigenic molecule (VaxiJen score: 0.6613, ANTIGENpro: 0.838802), while no allergenic, MEME/MAST motifs and IgE epitopes were found within the sequence. A significantly high protein solubility was estimated for NcIMP-1, with a Protein-Sol score of 0.764. The MW of the NcIMP-1 was 42.71 kDa (those proteins over 5-10 kDa are potent immunogens) [37], which is beneficial for SDS-PAGE and western blot analyses. An instability index of over 40 renders the protein to be unstable, as substantiated by instability score of 41.29. Moreover, this protein was highly thermotolerant in a wide range of temperatures (aliphatic index: 85.83) and showed to be hydrophilic in nature (GRAVY score: -0.447). The speculated pI for this protein was estimated as relatively acidic in nature (5.43), being advantageous for purification purposes in ion-exchange chromatography and isoelectric focusing. Altogether, such preliminary information may be required for future wet studies using NcIMP-1. With 33 regions, phosphorylation was the predominant PTM site in NcIMP-1 protein, followed by O-glycosylation (13 regions) and palmitoylation sites (one region). It is noteworthy that there was no N-glycosylation site in this protein. In total, these PTM regions are crucial in the recombinant production process of the proteins, so that eukaryotic expression systems (yeast, insect, or mammalian) are more preferred in comparison to bacterial hosts [35]. The presence of a signal peptide demonstrates that a synthesized protein could be destined towards several pathways, including excretory-secretory, virulence factor, or surface proteins [38]. Accordingly, based on the results from Signal-3L and SignalP web servers, no signal peptide was present in the NcIMP-1 sequence. The PSIPRED server demonstrated that extended strands and helices were the most prevalent secondary structures in this protein. Inevitably, the protein conformation is maintained and protected during molecular interactions using such internally located structures [39]. Disordered proteins are highly abundant, mostly dedicated to regulatory functions and molecular signaling. Supposedly, these regions are likely immunological targets for antibodies; hence, they seem to be important in vaccination studies [40]. However, no intrinsically disordered regions were predicted in the sequence. For 3D homology modelling, the SWISS-MODEL server was employed, which predicted a monomer model with high coverage and 23.03% identity. This model was further subjected to refinement and validations. Based on the ERRAT, Prosa-Web, and PROCHECK analyses, it was shown that the quality of the refined model was enhanced after refinement, in comparison with the crude model.

During *N. caninum* infection, both antibody-dependent and cellular immunity are recalled. Little is known on the possible role of B cell responses in protection [41]. It is plausible that antigen-specific antibodies, rather than polyclonal antibodies, inhibit tachyzoites from host cell invasion [42]. During the first 2 weeks, a significant increase in splenic B cells would occur, which regresses later on [43]. It was, also, found that B cell-depleted mice succumb to the infection 29 days postinfection [44]. Other prominent features of immunity against tachyzoite multiplication and bradyzoites reactivation are CTL (T CD_8_^+^) responses and the production of IFN-*γ* from both T CD_4_^+^ and T CD_8_^+^ cells [45, 46]. Nevertheless, not the whole sequence of a given protein shows affinity to these immunological cells. Based on this, several web servers were employed in the present study to accurately predict and screen the potential immunogenic epitopes in NcIMP-1. Although cattle is the target species for vaccination studies against neosporosis, mouse models are more accessible and affordable for such purposes (Aguado-Martínez et al., 2017); accordingly, we premised our immunoinformatics analyses on mouse MHC-I and MHC-II binding epitopes. A multistep approach was conducted to screen linear B cell epitopes using six web servers, three for identification of shared epitopes (BCPREDS, ABCpred, and SVMTriP) and three for screening phase (VaxiJen, AllerTOP, and PepCalc). Six epitopes qualified to be potentially immunogenic, including “VTEDGDVIVAVDE,” “TADSSKGRNSESK,” “MKYEQKGGKTE,” “KSIKGEKTNIV,” “STADSSKGRN,” and “EKAGKILVSFVPA.” Conformational B cell epitopes, also, have a remarkable role in the quality of antigen-antibody interactions. Thereby, we predicted these epitopes in the NcIMP-1 protein. The results showed 4 conformational epitopes by the length of 21, 24, 49, and 8 residues, respectively, and qualifying scores of 0.712, 0.697, 0.662, and 0.557. Furthermore, since antigen presentation is highly important for T cell priming, those epitopes with specific affinity to bind mouse MHC molecules were predicted using the IEDB server. With respect to MHC-I binding epitopes, four peptides were shown to be highly antigenic, nonallergenic, and nontoxic, including “VDLSVFSHVAVV,” “EEEKAGKILVSF,” “LPRDRPVDLSVF,” and “DEYEATLCVRNW.” About half of the predicted MHC-II binding peptides were IFN-*γ* inducers, while they failed to show above-threshold antigenicity scores. With the exception of one epitope (DLSVFSHVAVVPADK), all MHC-II binding epitopes were potent IL-4 inducers. Notably, only one CTL epitope (QVKATGGPV) passed the threshold of antigenicity score and was nonallergenic. Altogether, all of these epitopes could be further supplied in the multiepitope vaccine constructs and/or diagnostic polypeptides and evaluated in the context of wet experimental methods. The efficacy of epitope selection and screening procedures could be further improved in future studies in order to achieve more accurate, overlapping, and immune-dominant epitopic regions in examined proteins. For instance, ([47, 48] provided several steps for the prediction of candidate epitopes. Although, no screening was done in comparison with our study, they utilized a multimethod approach for the prediction of MHC-I and MHC-II binding epitopes. Also, they predicted overlapping T cell- and IFN-*γ*-inducing epitopes in examined proteins. They, also, utilized a cross-validating method for linear B cell epitope prediction (ABCpred, BcePred, and antibody-epitope prediction of IEDB), similar to our study which ABCpred, BCPREDS, and SVMTriP web servers were used and overlapping epitopes were selected and further screened regarding antigenicity, allergenicity, and water solubility.

## 5. Conclusion

*Neospora caninum* infection is a global threat to the cattle industry by inflicting reproductive failure and endemic/epidemic abortions. Therefore, there is an increasing need to recognize novel vaccine candidates to be used in the context of unprecedented immunization platforms. The interdisciplinary branch of science, bioinformatics, assists us to characterize the physicochemical features of a protein, to spot highly immunodominant epitopic regions, and to engineer a more rational vaccine design. The present *in silico* study highlighted the most important biophysical characteristics and novel B cell, MHC binding, and CTL epitopes of NcIMP-1 protein using a set of immunoinformatics servers, which could be directed towards immunization studies alone or combined with other dominant *N. caninum* antigens.

## Figures and Tables

**Table 1:** The final screening of shared linear B cell epitopes from *N. caninum* immune mapped protein-1 (IMP-1).

Shared B cell epitopes	VaxiJen antigenicity score	AllergenFP allergenicity prediction	PepCalc water solubility prediction
VTEDGDVIVAVDE^∗^	1.0899	No	Good
TADSSKGRNSESK^∗^	1.5902	No	Good
MKYEQKGGKTE^∗^	1.5936	No	Good
KSIKGEKTNIV^∗^	1.2111	No	Good
VSPRDLELLRQA	-0.0859	No	Good
AVAVAEGLQTAQK	0.1016	Yes	Good
STADSSKGRN^∗^	0.9542	Yes	Good
EKAGKILVSFVPA^∗^	0.6490	Yes	Good

^∗^Shared epitopes with high antigenicity, good water solubility, and nonallergenicity.

**Table 2:** Specific B cell linear epitopes of *N. caninum* immune mapped protein-1 (IMP-1) based on different physicochemical parameters predicted by the BcePred web server.

Physicochemical parameter	B cell epitopes
Hydrophilicity	CSKSTADSSKGRNSESKATEAERA, AVTEDGDV, AVDEEDKKSIKG, SRSPEPA, VTEEGKNGDTRV, TDVTNSDGQNRREYK, VPDKGGS, SPEEEEKAGK, KYEQKGGKTE, KANEKQKY, KTCDEYEAT, PADKSKEFK, SEKKFQD, DISDNKQGL

Flexibility	GVCSKSTADSSKGRNSESKA, VAVDEEDKKSIKGEK, AAVVSRSP, EVVTEEGKNGD, TDVTNSDGQNR, LYVPDKGG, QELSPEEEEKA, MKYEQKGGK, SVWKANEK, AVVPADKSKEF, EGHDLSEKK, IDISDNK, LILDGKK

Accessibility	SKSTADSSKGRNSESKATEAERA, QTAQKIE, AVDEEDKKSIKGEKTNIVDE, VSRSPEPAAVIRKEEVVTEEGKNGDTRV, DLPEQVEHVSPRD, ELLRQAREQVKAT, TDVTNSDGQNRREYKPRASAY, LKQELSPEEEEKAGKI, HKSVPRMKYEQKGGKTEL, VWKANEKQKYYAAW, KTCDEYEATL, VRNWTEDMPPQV, ATLPRDRPVDL, VPADKSKEFKEGHDLSEKKFQDL, HQRLVPRGNAAK, DISDNKQGL

Turns	DVTNSDGQ

Exposed surface	VDEEDKKSIKGEK, QAREQVK, DGQNRREYKPRAS, KQELSPEEEEKAGK, KSVPRMKYEQKGGK, WKANEKQKYYAA, PRDRPVD, PADKSKEFKE, HDLSEKKFQDL, LDGKK

Polarity	SKGRNSESKATEAERAV, VAVDEEDKKSIKGEKTNI, AVIRKEEVVTEEGKNGD, LPEQVEHVSPRDLELLRQAREQVKAT, SDGQNRREYKPRAS, KQELSPEEEEKAGKI, LHKSVPRMKYEQKGGKTE, VWKANEKQKYYA, KTCDEYE, PRDRPVDL, PADKSKEFKEGHDLSEKKFQDL, HQRLVPR, FKEDGIDI, DGKK

Antigenic propensity	TRVHVSVVE, PEQVEHVSPR, GPVITDVT, YGYLLYVPDKGGSLILLWLKQ, GKILVSFVP, LHKSVPR, TVLKTCD, MPPQVFLSLLHVGPVG, RPVDLSVFSHV

**Table 3:** Prediction of mouse MHC-I binding epitopes of *N. caninum* immune mapped protein-1 (IMP-1) using IEDB server followed by antigenicity, allergenicity, and toxicity screening.

Mouse MHC-I alleles	Position	T cell peptide	Percentile rank	VaxiJen antigenicity score	AllergenFP allergenicity prediction	ToxinPred toxicity prediction
H2-Db	12-23	VEHVSPRDLELL	2.9	0.1894	Yes	Nontoxin
	24-35	VVSRSPEPAAVI	4.8	0.2167	No	Nontoxin
	4-15	YVPDKGGSLILL	5.6	-0.8740	No	Nontoxin
	46-57	GAPAAVTEDGDV	5.8	1.2707	Yes	Nontoxin
	19-30	VAVVPADKSKEF	6.8	0.4950	No	Nontoxin

H2-Dd	4-15	YVPDKGGSLILL	1.1	-0.8740	No	Nontoxin
	12-23	VEHVSPRDLELL	1.5	0.1894	Yes	Nontoxin
	24-35	VVSRSPEPAAVI	2.0	0.2167	No	Nontoxin
	41-52	TEDMPPQVFLSL	2.6	1.0264	Yes	Nontoxin
	1-12	YLLYVPDKGGSL	2.9	-0.0065	No	Nontoxin

H2-Kb	4-15	YVPDKGGSLILL	6.2	-0.8740	No	Nontoxin
	29-40	AGKILVSFVPAL	8.2	0.6598	Yes	Nontoxin
	11-22	VDLSVFSHVAVV^∗^	9.1	0.6512	No	Nontoxin
	36-47	FVPALHKSVPRM	11	-0.1124	Yes	Nontoxin
	41-52	TEDMPPQVFLSL	11	1.0264	Yes	Nontoxin

H2-Kd	14-25	KQKYYAAWATVL	1.4	0.4647	Yes	Nontoxin
	48-59	KYEQKGGKTELL	1.9	0.9165	Yes	Nontoxin
	3-14	LYVPDKGGSLIL	2.0	-0.6096	No	Nontoxin
	39-50	NWTEDMPPQVFL	3.4	1.7663	Yes	Nontoxin
	2-13	LLYVPDKGGSLI	7.6	-0.4493	Yes	Nontoxin

H2-Kk	41-52	TEDMPPQVFLSL	0.87	1.0264	Yes	Nontoxin
	20-31	SKATEAERAVAV	1.6	1.0536	Yes	Nontoxin
	18-29	SESKATEAERAV	2.0	1.0414	Yes	Nontoxin
	25-36	EEEKAGKILVSF∗	2.2	1.1575	No	Nontoxin
	29-40	DEYEATLCVRNW	2.2	1.1189	No	Nontoxin

H2-Ld	41-52	TEDMPPQVFLSL	0.61	1.0264	Yes	Nontoxin
	44-55	MPPQVFLSLLHV	1.1	0.6408	No	Nontoxin
	5-16	LPRDRPVDLSVF∗	1.3	0.9249	No	Nontoxin
	5-16	VPDKGGSLILLW	1.5	-0.9925	Yes	Nontoxin
	19-30	VAVVPADKSKEF	1.5	0.4950	No	Nontoxin

H-2-Q1	4-15	YVPDKGGSLILL	3.0	-0.8740	No	Nontoxin
	7-18	SVWKANEKQKYY	3.9	0.2189	No	Nontoxin
	41-52	VTNSDGQNRREY	4.1	0.3669	No	Nontoxin
	1-12	YLLYVPDKGGSL	5.7	-0.0065	No	Nontoxin
	10-21	KANEKQKYYAAW	5.9	0.5640	No	Nontoxin

H-2-Q2	41-52	TEDMPPQVFLSL	0.78	1.0264	Yes	Nontoxin
	38-49	RNWTEDMPPQVF	1.1	1.2711	No	Nontoxin
	27-38	REQVKATGGPVI	1.3	0.9078	Yes	Nontoxin
	25-36	EEEKAGKILVSF	1.3	1.1575	No	Nontoxin
	29-40	DEYEATLCVRNW∗	1.4	1.1189	No	Nontoxin

^∗^Potential qualified epitopes.

**Table 4:** Prediction of mouse MHC-II binding epitopes of *N. caninum* immune mapped protein-1 (IMP-1) using IEDB web server followed by screening for antigenicity, allergenicity, and IFN-*γ*/IL-4 induction.

Mouse MHC-II alleles	Position	T cell peptide	Percentile rank	VaxiJen antigenicity score	AllergenFP allergenicity prediction	IFN-*γ* induction	IL-4 induction
H2-IAb	13-27	EKQKYYAAWATVLKT	0.5	0.0589	No	Positive	Positive
	12-26	NEKQKYYAAWATVLK	0.58	0.1720	Yes	Positive	Positive
	11-25	ANEKQKYYAAWATVL	0.72	0.4536	No	Positive	Positive
	14-28	KQKYYAAWATVLKTC	0.8	0.1605	No	Positive	Positive
	15-29	QKYYAAWATVLKTCD	0.91	-0.0727	Yes	Positive	Positive
	39-53	QKIEAVTGAPAAVTE	0.94	0.3219	No	Positive	Positive

H2-IAd	13-27	EKQKYYAAWATVLKT	1.62	0.0589	No	Positive	Positive
	14-28	KQKYYAAWATVLKTC	1.75	0.1605	No	Positive	Positive
	15-29	QKYYAAWATVLKTCD	2.10	-0.0727	Yes	Positive	Positive
	12-26	NEKQKYYAAWATVLK	2.18	0.1720	Yes	Positive	Positive
	13-27	LSVFSHVAVVPADKS	2.77	0.4540	No	Negative	Positive
	12-26	DLSVFSHVAVVPADK	3.2	0.5379	No	Negative	Negative

H2-IEd	35-49	SFVPALHKSVPRMKY	1.5	0.0715	No	Positive	Positive
	34-48	VSFVPALHKSVPRMK	2.05	0.0013	No	Positive	Positive
	36-50	FVPALHKSVPRMKYE	2.35	0.1416	No	Positive	Positive
	2-16	IEDKWSVWKANEKQK	2.6	0.2682	No	Negative	Positive
	13-27	EKQKYYAAWATVLKT	2.75	0.0589	No	Positive	Positive
	14-28	KQKYYAAWATVLKTC	2.9	0.1605	No	Negative	Positive

**Table 5:** Prediction of top ten cytotoxic T lymphocyte (CTL) epitopes of *N. caninum* immune mapped protein-1 (IMP-1) using CTLPred web server with antigenicity, allergenicity, and hydrophobicity screening.

Rank	Start position	Peptide sequence	Score (ANN/SVM)	VaxiJen antigenicity score	AllergenFP allergenicity prediction	Hydrophobicity (%)
1	124	DLADLPEQV	0.93/1.0930182	-0.5829	Yes	55.56
2	230	EQKGGKTEL	0.93/0.94425972	0.6914	Yes	11.11
3	86	SRSPEPAAV	0.50/1.3343562	0.1781	No	55.56
4	356	VPRGNAAKL	1.00/0.80563294	0.4124	No	55.56
5	23	TEAERAVAV	0.85/0.93975112	0.9334	Yes	55.56
6	76	IVDEAAAAV	0.76/0.85800815	0.3109	Yes	77.78
7	303	ATLPRDRPV	0.41/1.1165353	1.1887	Yes	55.56
8	267	TCDEYEATL	0.95/0.56175719	1.2626	Yes	22.22
9	149	QVKATGGPV^∗^	0.16/1.2059993	0.9845	No	44.44
10	273	ATLCVRNWT	0.95/0.37756272	1.0445	Yes	44.44

^∗^Qualified epitope.

## Data Availability

The data used to support the findings of this study are included within the article.
